# Official health communications are failing PFAS-contaminated communities

**DOI:** 10.1186/s12940-022-00857-9

**Published:** 2022-05-11

**Authors:** Alan Ducatman, Jonas LaPier, Rebecca Fuoco, Jamie C. DeWitt

**Affiliations:** 1 School of Public Health, West Virginia University, Morgantown, WV USA; 2 Green Science Policy Institute, Berkeley, CA USA; 3 Brody School of Medicine, East Carolina University, Greenville, NC USA

**Keywords:** Per- and polyfluoroalkyl substances (PFAS), Health communication, Fluorocarbons, Shared decision-making, Secondary prevention

## Abstract

**Background:**

Environmental health agencies are critical sources of information for communities affected by chemical contamination. Impacted residents and their healthcare providers often turn to federal and state agency webpages, fact sheets, and other documents to weigh exposure risks and interventions.

**Main body:**

This commentary briefly reviews scientific evidence concerning per- and polyfluoroalkyl substances (PFAS) for health outcomes that concern members of affected communities and that have compelling or substantial yet differing degree of scientific evidence. It then features official documents in their own language to illustrate communication gaps, as well as divergence from scientific evidence and from best health communication practice. We found official health communications mostly do not distinguish between the needs of heavily contaminated communities characterized by high body burdens and the larger population with ubiquitous but substantially smaller exposures. Most health communications do not distinguish levels of evidence for health outcomes and overemphasize uncertainty, dismissing legitimate reasons for concern in affected communities. Critically, few emphasize helpful approaches to interventions. We also provide examples that can be templates for improvement.

**Conclusions:**

Immediate action should be undertaken to review and improve official health communications intended to inform the public and health providers about the risks of PFAS exposure and guide community and medical decisions.

**Supplementary Information:**

The online version contains supplementary material available at 10.1186/s12940-022-00857-9.

## Introduction

Health communications concerning environmental risk, risk avoidance, and risk mitigation are challenging. Challenges include public and health professional unfamiliarity with the topic material, the difficult balance between conveying an actionable message without engendering unneeded fear, as well as perceived disconnections between the language used by medical and public health professionals and the public they serve [1]. This article evaluates official health communications on per- and polyfluoroalkyl substances (PFAS) and provides recommendations for making these communications more science-based and effective.

PFAS are a class of thousands of synthetic chemical compounds used in industrial products such as firefighting foams and lubricants as well as consumer products such as coated fabrics, carpets, cookware, food packaging, and many other applications [2]. Individual PFAS may also be by-products of commercially relevant PFAS uses, which include transformation products of precursor compounds as well as breakdown products from consumer and industrial goods. Because of widespread use, as well as their mobility and persistence, most humans have detectable internal PFAS contamination from multiple sources, notably food, food contact materials, and indoor products [3]. Near-ubiquitous “background” levels of blood contamination have been detected in a United States nationally-representative population. These findings and how the levels have changed over time can be found in the comprehensive tables of the Centers for Disease Control and Prevention (CDC) “National Report on Human Exposure to Environmental Chemicals” [4].

However, an unknown number of people around the world estimated to include millions in the United States alone [5–7], have endured more substantial internal exposures, notably from work exposure or drinking water contamination. These include community drinking water exposures to PFAS at levels higher (and sometimes far higher) than the current US Environmental Protection Agency (EPA) PFAS drinking water health advisory of 70 ng/L for two “legacy” PFAS, perfluorooctanoic acid (PFOA) and perfluorooctane sulfonic acid (PFOS), leading to higher measurable serum contamination. Communities with striking levels of human PFAS exposure from drinking water include Arnsberg, Germany [8], contiguous areas in West Virginia and Ohio in the United States [9], the Veneto region of Italy [10], residents near a Science Park in Taiwan [11], and Ronneby, Sweden [12]. The US National Institute for Occupational Safety and Health (NIOSH) also notes that occupational populations have high exposure levels [13], and health outcomes have been reported from those populations as well [14–18].

Furthermore, based partially on the known toxicity of PFOA, PFOS, and similar PFAS compounds that have long half-lives in humans, a growing array of replacement PFAS compounds have been introduced for industrial or consumer use. Some of these already contaminate water supplies. The experience of the Cape Fear River region of North Carolina illustrates how a population historically exposed to well-known legacy PFAS such as PFOA or PFOS may subsequently face water contamination by newer or less well-known compounds [19]. Some of these less studied PFAS are even harder to remediate for acceptable drinking water once contamination has occurred [2, 19, 20].

Operators of small water systems, private well owners, and affected communities can have inadequate resources for obtaining alternative water supplies or for installation and long-term maintenance of PFAS water remediation equipment [21]. The environmental justice problem of scarce resources and significant remediation costs can drive a wedge between the economic interests of local officials or water managers and the public health interests of affected communities. Private well owners may face decisions involving both remediation costs and health concerns. Community members affected by substantial PFAS exposure have reported seeking preventive health care services yet also report significant hurdles in obtaining resources [22].

Agencies and organizations providing health communications are responsible for preventing exaggeration or wording that engenders undue fear. Conversely, official health communications that unrealistically minimize the health risks in high exposure circumstances might increase the hurdles faced by heavily affected populations seeking guidance for exposure remediation or health mitigation strategies. In recent open meetings sponsored by the National Academies of Sciences, Engineering, and Medicine [22], community members from PFAS-affected communities articulated a repeated perception that health communications intended to assist them actually provide unreasonably-minimizing scientific language concerning addressable outcomes. They described the health, economic, and social effect on their lives, including dismissive reactions by policy makers or healthcare providers that are sometimes influenced by official health communications.

## Background

This work reviews how health communications serve communities with high internal PFAS contamination. To provide a background for comparison, it begins with a brief review of the literature on PFAS health outcomes. A more thorough accounting of the research on PFAS health outcomes is available from the PFAS-Tox Database [23]. The bulk of evidence is for perfluorooctanoic acid (PFOA) and perfluorooctane sulfonic acid (PFOS), which are the most prominent PFAS among highly exposed populations described in peer-reviewed studies to date. Substantial but less evidence exists for some other PFAS compounds, such as perfluorohexane sulfonic acid (PFHxS) and perfluorononanoic acid (PFNA).

We limited our evidence review to health outcomes that affected communities and clinicians have voiced the most concern about, and that also have a compelling or substantial evidence basis, but recognize that others could have been considered. We sought articles related to the following: breast feeding, cancer (kidney cancer and testicular cancer), immune, and adult liver/lipid outcomes. Synonyms or closely related concepts included breastmilk, cholesterol, renal, steatosis, transaminases, and uric acid, and their intersection with PFAS, PFOA, and PFOS in the US National Library of Medicine and Web of Science.

We attempted to take the main points about emerging consensus from recent review articles and official organizational sources concerning outcomes, including primary sources to illustrate specific points from reviews to assist readers with topics that directly illustrate weight-of-evidence concepts.

We also searched for health communications documents intended for the lay public or for clinicians. These are from cognizant state and federal and international government agencies, professional societies, and other authoritative groups in the United States and abroad, using agency organization names and PFAS, PFOA, and PFOS keywords in Google and Google Scholar. We found many such documents and limited our tabular reporting to those intended for health communications to the lay public or to healthcare providers. A limitation of this approach is that the search was in English, and the numerous sites are updated unpredictably so our data reflect what was publicly available at the time we performed the search. The sites that form the basis of citations in this document were last accessed between March 7–30, 2022.

### PFAS health outcomes

#### Immune

The US National Toxicology Program has concluded that PFOA and PFOS are “presumed to be an immune hazard to humans” [24]. The Human Biomonitoring Commission of the German Environment Agency determined that adverse outcomes with corroborative human evidence include diminished vaccine response and increased frequency, severity, and chronicity of infectious diseases [25]. The US Environmental Protection Agency (EPA) recently drafted proposed reference doses for PFOA and PFOS that are based upon lowered human antibody responses to administered vaccines, noting that human epidemiological studies are generally consistent with an association between PFOA or PFOS exposure and immunosuppression in children [26]. The US state of New Jersey also utilized immune suppression for their PFOS reference dose determination, citing human population data while using parallel experimental animal data for their derivation [27]. The case for PFAS alterations in vaccine response is compelling. PFAS-associated alterations in immune responses may also affect organ function more broadly, with substantial human and experimental evidence for immunotoxic mechanisms contributing to adverse lung and liver function [28].

#### Liver and lipids

PFAS-associated abnormalities of liver toxicity are reflected in higher liver blood enzyme values such as alanine transaminase (ALT) values, and altered lipid metabolism. Multiple adult human population studies also show alterations in liver functions associated with PFAS exposure, and notably with PFOA, including all studies in large populations with a wide range of exposure levels [28]. For example, a first to fifth quintile increase in cumulatively modeled PFOA exposure in the C8 population resulted in a 16% increase in above-normal ALT findings [29], a finding of clinical biomarker abnormality that was not considered to be attributable to reverse causation in follow-up research [30].

In studies designed to evaluate associations between PFAS exposure and hepatotoxicity, additional clinically diagnosed liver disease was not noted in 3 years of follow-up [29]. However, the American College of Gastroenterology clinical guidelines note that regardless of inciting cause, higher ALT is reliably associated with increased liver disease and mortality [31]. Higher ALT is also a population risk factor for hepatic, cardiovascular, and infectious disease morbidity and mortality [31–36]. Conversely, lower ALT is one of the predictors of improved nonalcoholic fatty liver disease (NAFLD) activity in clinical trial settings [37].

For PFAS and liver toxicity, there are parallel experimental data. PFAS reliably disrupt liver metabolism in experimental animal models, leading to lipid droplet infiltration, enlargement of hepatocytes, evidence of steatosis in cell lines and across experimental species, as well as elevation of liver function enzymes that mark hepatocellular damage such as ALT [28, 38–43].

Steatosis is the first step in a chain of events that can lead to nonalcoholic fatty liver disease (NAFLD), which has stages from early subclinical disease to inflammatory disease, fibrosis/cirrhosis, and death. In humans, the progression of NAFLD is accompanied by disrupted lipid metabolism and proatherogenic lipid profiles [44]. Unsurprisingly, > 20 human study populations concerning PFAS outcomes (and many more than 20 animal studies) reveal an additional outcome of hepatotoxicity - adverse total cholesterol and low-density lipoprotein (LDL) cholesterol and apolipoprotein outcomes, notably but not exclusively for PFOA and PFOS [28]. The association of PFAS exposure to higher cholesterol has been found in cross-sectional, case-control, and longitudinal studies, with some longitudinal studies nested in clinical trials for populations with preexisting hepatic metabolism susceptibilities [28, 45, 46]. Large population studies with sufficient ranges of exposure reveal a replicable cholesterol dose response [47, 48]. Increased diagnosable hyperlipidemia in association with PFAS exposure is also present in adolescence [49–51], rendering hypotheses about medication effects unlikely. Experimental animal data that support and likely explain the human outcomes have been published, including histologic findings of steatosis across animal species. A variety of metabolic pathways have been implicated for PFAS-disrupted lipid metabolism [28, 52–55]; a remaining challenge is to determine which pathways are most important. Noncausal explanations for the liver and lipid population findings and even experimental findings have been extensively considered as reviewed in Anderson et al. [56, 57]. However, so far no convincing demonstration of such confounding exists. Instead there is a developing consensus that PFAS are a source of human hepatoxicity including disrupted lipid metabolism [7]. Hypotheses about so far undetected sources of human population confounding do not address the parallel human and experimental biomarker findings and relevant pathways in experimental studies, including parallel findings of disrupted liver transaminase, lipid, and uric acid metabolism [39, 58–64].

Authors from the European Food Safety Authority (EFSA) have noted that PFOA, PFOS, and PFNA are associated with worsened lipid profiles [46]. The US EPA draft review has characterized the human associations of PFAS exposure to disrupted lipid metabolism as “robust and consistent [26].” The German Human Biomonitoring Commission determined that PFAS exposure detrimentally alters lipid metabolism in humans [25]. Considering the human lipid and liver enzyme data, and the experimental data with parallel evidence of liver enzyme abnormalities and steatosis across species, there is abundant, compelling evidence that PFAS are hepatoxic.

#### Breastfeeding

Breastfeeding is inherently advantageous for both maternal and child health, universally recommended, and important to parents and children. Well-designed studies reveal that PFAS exposure is associated with a diminished ability to breastfeed or early termination of breastfeeding at elevated PFAS exposures, as reviewed in the EPA draft document concerning PFOA [26].

One group hypothesized that the association is due to reverse causation because they found the association to be less clear in primiparous compared to multiparous women [65]. However, independent researchers have found the association in both multiparous and primiparous women and in longitudinal data [66, 67], so it is unclear how reverse causation, which should not pertain to nulliparous women, could be a unitary explanation for associations. Human population evidence for the role of PFAS in decreasing breastfeeding has been augmented since the EPA draft review [26]. PFOS, PFOA, PFNA and summed PFAS were prospectively associated with increased risk of termination of breastfeeding, an association that strengthened after adjustment for confounders [68]. In the Ronneby cohort, mothers who were heavily PFAS-exposed had higher risk of not initiating breastfeeding and of shorter duration of breastfeeding, with stronger decrements in primiparous mothers. The authors interpreted the data to show that breastfeeding is a sensitive outcome of PFAS exposure in primiparous women [69]. The data concerning breastfeeding duration are complemented by laboratory evidence that PFAS exposure during pregnancy is prospectively associated with decrements in human breast milk quality following delivery [70].

Experimental studies provide supportive data. PFAS exposure reliably affects mammary gland development in experimental settings, reviewed in support of drinking water guidance in the New Jersey [71]. PFAS exposure upregulates PPAR gamma nuclear receptor pathways that may interfere with breast development and health, and PFAS suppress protein coding genes known to be important to mammary gland development [72, 73].

#### Testicular and kidney cancer

The International Agency for Research on Cancer (IARC) classified PFOA as a possible human carcinogen based on testicular and kidney cancer data (Class 2B); a summary can be found in [74] . The recent EPA draft document for PFOA characterizes the weight of evidence as supportive of a carcinogenic effect of PFOA [26]. The conclusion is based on findings of kidney and testicular cancer in cohort, case-control, and cross-sectional cancer studies. The EPA draft also noted the supportive evidence of carcinogenicity from experimental studies, including recently added studies of multiorgan tumorigenesis in animal studies. The most recent review in the peer-reviewed literature (from authors in California, Nevada, and North Carolina state agencies) concluded that PFOA is a likely cause of both kidney and testicular cancer in humans [75].

There are several other recent reviews of PFAS carcinogenicity [76, 77]. A case-control study in China found that six of ten measured serum PFAS were significantly associated with pediatric germ cell tumors [78]. The most compelling human study is a case-control comparison nested within a clinical trial by US National Institutes of Health authors [79]. This work yielded 324 cases in a population of ~ 150,000 after ≥8 years of longitudinal follow up and detected a dose-response across quartiles of internal PFOA exposure and a statistically significantly greater than twofold risk of kidney cancer in the highest vs. the lowest quartile of PFOA [79]. The EPA draft and other reviews note that mechanisms are likely nongenotoxic and probably related to membrane receptor activation, endocrine disruption, and epigenetic alterations [80].

In their review of PFOA and as a specific cause of kidney and testicular cancer, Bartell and Vieira noted that the associations are both most likely causal and also that the evidence regarding testicular cancer has remained more sparse than the evidence for kidney cancer [81]. However, consistent experimental findings of alterations in steroidogenesis in testicular cells, and damage via endocrine disruption and estrogen receptor signaling indicate that the testes are target organs [82–84]. Further, PFAS mixtures have negative effects on testicular stromal cells in humans [85] and possibly on testicular volume [86].

Substantial data support a role of PFAS in human carcinogenicity. The human population evidence is strongest for kidney cancer and supportive for testicular cancer.

### Review of official health communications on PFAS

Table 1 highlights main messages by national, state, and local agencies, as well as nongovernmental and professional organizations about PFAS that are intended for the public or for clinicians. Supplemental Table One (Table S1) lists main messages, quotes, and links to URLs. Most are from the United States. Many discuss drinking water contamination. Examples are provided to illustrate common literature gaps, poorly conceived communications, or useful examples that can provide templates for improvement.

**Table 1:** Excerpts from Official Health Communications concerning PFAS

Agency/ Organization	Short Excerpt
**US Federal Agencies**	
Agency for Toxic Substances and Disease Registry	A large number of studies have examined possible relationships between levels of per- and polyfluoroalkyl substances (PFAS) in blood and harmful health effects in people. However, not all of these studies involved the same groups of people, the same type of exposure, or the same PFAS. These different studies therefore reported a variety of health outcomes. Research involving humans suggests that high levels of certain PFAS may lead to the following …
ATSDR Clinician Guidance on PFAS	It is possible that PFAS contributed to your health problems but there is no way to know if PFAS exposure has caused your illness or made it worse … Based on what we know at this time, there is no reason to think your health problem is associated with exposure to PFAS.
ATSDR Talking to Your Doctor about Exposure to PFAS	… some studies in people have shown that certain PFAS may affect growth, learning, and behavior of infants and older children, lower a woman’s chance of getting pregnant, interfere with the body’s natural hormones, increase cholesterol levels, affect the immune system, increase the risk of cancer. If you have any of these conditions and have been exposed to PFAS, you can tell your doctor.
Department of Defense	Scientists are still studying the health effects of exposure to PFAS. Although more research is needed, some studies in people have shown that certain PFAS may affect health.
Environmental Protection Agency	Because certain PFAS are known to cause risks to human health, the most important steps you and your family can take to protect your health is to understand how to limit your exposure to PFAS.
Food and Drug Administration	Exposure to some types PFAS have been linked to serious health effects.
National Institute of Environmental Health Sciences	The research conducted to date reveals possible links between human exposures to PFAS and adverse health outcomes.
National Toxicology Program	NTP is studying the potential health effects of PFAS through a large research effort with multiple facets including experimental rodent and cell-based test systems, literature review, and computer modeling, among others. Taken together, these studies give insights into the potential adverse health outcomes of PFAS in the human body.
**Foreign Agencies**	
Australian Government Department of Health	PFAS has not been proven to cause any specific illnesses in humans …There is no current evidence that supports a substantial impact on an individual’s health from PFAS exposure.
Australian Government National Health and Medical Research Council (Recreational Water)	PFAS has not been shown to cause disease in humans.
European Chemicals Agency	Certain PFASs are known to accumulate in living things and cause toxic effects.
European Environmental Agency	See Fig. 1 for portrayal of health effects with denotation of high and low uncertainty
Government of Canada	Adverse environmental and health effects have been observed for well-studied PFAS (PFOS, PFOA, and LC-PFCAs and their salts and precursors) and they have been shown to pose a risk to the Canadian environment … PFAS used to replace regulated PFOS, PFOA, and LC-PFCAs may also be associated with environmental and/or human health effects.
**NGOs / Professional Organizations**	
American Academy of Pediatrics	We currently do not have a clear answer on how PFAS can impact health but there are scientific studies going on right now to help us answer this question. Some of these studies show a possible connection between PFAS exposure and higher cholesterol levels, as well as effects on the hormone system, immune system, liver, and kidneys.
American Association for the Advancement of Science - Risk Communications for Local and State Leaders	A class of thousands of synthetic organic chemicals, not enough is known about the health impacts of most PFAS, but even small doses of several of the most-researched compounds can lead to health issues. These guides can help people engage their community members, drinking water providers, local and state regulatory agencies, and federal agencies to address PFAS in drinking water.
American Water Works Association	The EPA and the Agency for Toxic Substances and Disease Registry (ASTDR) both report that the most consistent health effect from PFAS exposure is increased cholesterol levels. There are more limited findings related to [list of health effects].
Association of State and Territorial Health Officials - Clinicians FAQ	There is evidence linking certain PFAS to adverse health effects in humans, with more evidence for some effects than for others.
Association of State and Territorial Health Officials - General FAQ	Studies show that different PFAS can cause different types of toxicity; EPA classifies them as having “suggestive” evidence of human carcinogenicity.
Association of State and Territorial Health Officials - Public FAQ	It is difficult to link current health issues with PFAS exposure.
Environmental Council of the States - Health Communications Case Studies	See health risk communications case studies
Green Science Policy Institute	The best-studied PFAS, PFOA and PFOS, are linked to liver damage, high cholesterol, obesity, diabetes, cancer, thyroid disease, asthma, immune system dysfunction, reduced fertility, low birth weight, and effects on children’s cognitive and neurobehavioral development. Ongoing research is finding similar health harm in other PFAS as they are studied.
Health and Environment Alliance	Scientific evidence suggests that exposure to PFAS can cause serious health impacts, among which kidney and liver damage, cancer, impaired fertility and immunity, and adverse pregnancy outcomes.
Interstate Technology Regulatory Council	Some PFAS have been linked to multiple health endpoints in studies of the general population and communities with contaminated drinking water. Laboratory animal toxicology studies and human epidemiological studies suggest health effects that may occur as a result of long-term exposure to PFOA and PFOS at environmentally relevant concentrations.
National Groundwater Association - PFAS Risk Communication for Contractors	There is evidence some PFAS can be harmful to human health.
National Resource Defense Council	Linked to a variety of health problems, among them cancer and developmental issues, PFAS can be harmful at extremely low doses.
Pediatric Environmental Health Specialty Units	While current evidence is compelling, causality has not been definitively established for a wide range of health effects. Many uncertainties and data gaps remain and will require further research. The most consistent findings from epidemiology studies are elevated blood serum total cholesterol levels among exposed populations, with strong evidence for a causal relationship between PFOA exposure and elevations in serum lipids.
Sierra Club	They are linked to a variety of health problems including kidney and testicular cancer, immune system damage, high cholesterol and digestive system problems, and significant changes to liver, thyroid, and pancreatic function.
Silent Spring Institute	Silent Spring Institute is studying this class of chemicals because some types of PFAS have been linked to cancers, including breast cancer, immunotoxicity in children, thyroid disease, reproductive problems, and other health effects.
Silent Spring Institute - PFAS REACH Information for Clinicians	See guidance for clinicians for performing laboratory tests, examinations, or counseling for adults and children
Silent Spring Institute - PFAS REACH Information for Patients	Several national and international health agencies have reviewed the results of peer-reviewed epidemiological (human populations) and toxicological (laboratory animals) research and written scientific assessments based on these studies, including [list of studies] … At least one of these assessments concluded that PFAS exposure is associated with [list of health effects].
**US States**	
Alabama Department of Environmental Management	Studies have shown possible adverse human health effects from exposure to PFAS.
Alaska Department of Spill Prevention and Response	Although these two compounds are the most studied, a growing body of research indicates additional PFAS compounds may have similar health or environmental effects and may be co-contaminants.
Arizona Department of Environmental Quality	PFAS exposure is linked to potential adverse human health outcomes …
Arkansas Department of Health	Some health studies indicate that exposure to PFOA and PFOS over certain levels may result in adverse health effects, including developmental effects to fetuses during pregnancy or to breastfed infants, cancer, liver effects, immune effects, thyroid effects, and other health effects.
California Environmental Protection Agency	Exposure to unsafe levels of PFOA/PFOS may result in adverse health effects including developmental effects to fetuses during pregnancy, cancer, liver effects, immune effects, thyroid effects, and other effects (such as cholesterol changes).
Colorado Department of Health and Environment	Whether PFAS chemicals harm health depends on many factors. These factors include amount of exposure, age, genetics, and health history. Research involving humans strongly suggests exposure to certain PFAS chemicals, including PFOA and PFOS may [list of health effects] … Though additional research is needed, it is likely other PFAS may have health impacts like PFOA and PFOS.
Connecticut Department of Energy and Environmental Protection	Certain PFAS have been linked to health risks including developmental effects in fetuses and infants, various forms of cancer, and decreased liver, thyroid, and immune system function.
Connecticut Department of Public Health	Therefore even low levels in drinking water may increase your risk of developing a variety of health risks if exposure is long term (months to years). Exposure to PFAS above the CT Action Level does not necessarily mean that health effects will happen.
Delaware Natural Resources and Environmental Control & Delaware Department of Health and Social Services	Some toxicological studies have found that exposure to these substances can cause serious health effects.
Florida Department of Environmental Protection	When released into the environment, PFAS can cause contamination to soil, groundwater and surface water, and these impacts may pose a risk to public health and the environment.
Georgia Environmental Protection Division	Peer-reviewed studies of laboratory animals and epidemiological studies of human populations indicate that exposure to PFOA and PFOS over certain levels may result in adverse health effects.
Hawaii Department of Health	While there are thousands of PFASs, only a relatively small number are considered to pose a significant risk to human health and the environment ... Some of the newer replacement compounds, such as HFPO-DA (GenX) and ADONA, are being evaluated for potential risks.
Illinois Environmental Protection Agency	While research on the effects of PFAS exposure on human health is ongoing, current scientific studies have identified possible adverse health effects such as increased cholesterol levels, increased risk for thyroid disease, low infant birth weights, reduced response to vaccines, liver and kidney toxicity, and pregnancy-induced hypertension.
Indiana Department of Environmental Management	Both PFOA and PFOS are commonly found in the environment. Studies indicate that exposure to PFOA and PFOS above certain levels may result in adverse health effects.
Iowa Department of Natural Resources	The existing body of scientific literature suggests that exposure to these compounds may result in health effects such as developmental defects in fetuses and infants as well as certain types of cancer.
Kentucky Energy and Environment Cabinet	There is evidence that exposure to PFAS may impact reproductive and developmental health, increase the risk for cancer, disrupt thyroid hormones, and affect the immune system.
Maine Department of Environmental Protection	Health agencies are working to understand more about the health effects of low level, long-term exposure.
Maryland Department of the Environment	According to the Agency for Toxic and Disease Registry (ATSDR) some, but not all, studies in humans with PFAS exposure have shown that certain PFAS may [list of health effects].
Massachusetts Department of Environmental Protection	Studies indicate that exposure to sufficiently elevated levels of certain PFAS may cause a variety of health effects including developmental effects in fetuses and infants, effects on the thyroid, liver, kidneys, certain hormones and the immune system. Some studies suggest a cancer risk may also exist in people exposed to higher levels of some PFAS.
Michigan Department of Environment, Great Lakes, and Energy	Health effects associated with PFAS include [list of health effects] … Studies in animals help us understand what could happen in people. Animals given very high amounts of PFOS and PFOA showed [list of health effects].
Minnesota Department of Health	Numerous studies have shown that higher levels of exposure to PFAS are associated with a wide range of human health effects … However, more work needs to be done to determine if PFAS, or other factors, caused the effects.
Minnesota Department of Health - Fact Sheet for Health Professionals	Drinking water at or above the guidelines does not pose an immediate health risk. We do not have evidence of human harm at current levels.
Montana Department of Environmental Quality	There is evidence that exposure to PFAS can lead to adverse human health effects.
New Hampshire Department of Health and Human Services	Available scientific research does not provide consistent information about whether PFAS cause health problems in humans … Because of inconsistent and contradictory findings of the effects of PFAS in people, more scientific studies are needed to be sure which health effects, if any, are caused by exposure to PFAS.
New Jersey Department of Health	Since human health effects are associated with even low-level exposures to PFOA and PFOS, it is important to minimize increases in exposure from drinking water.
New Mexico Environment Department	The health effects of these emerging contaminants are still being studied, but research indicates that some PFAS may affect reproductive health, increase the risk of some cancers, affect childhood development, increase cholesterol levels, affect the immune system, and interfere with the body’s hormones.
Rick Langley Letter to North Carolina Clinicians	It remains unclear if these tests [PFAS blood tests] would be clinically useful, and it is not possible to connect PFAS test results with clinical outcomes.
North Carolina Department of Health and Human Services	Whether or not you develop health problems after being exposed to PFAS depends on how much, how often, and for how long you are exposed, as well as which PFAS you are exposed to. Personal factors including age, lifestyle, and overall health can impact your body’s ability to respond to chemical exposures.
North Dakota Department of Environmental Quality	Some studies have shown a relationship between PFAS chemicals in the body and a higher chance of some diseases … Many but not all studies in humans show that certain PFAS chemicals may harm developing fetuses and cause problems during childhood development.
Ohio Environmental Protection Agency	Studies in humans and animals show that there may be negative health effects from exposure to certain PFAS. Completely stopping exposure to PFAS is not practical, because they are so common and present throughout the world.
Oregon Health Authority	The research suggests that exposure to high levels of PFAS may [list of health effects].
Pennsylvania Department of Environmental Protection	PFOA and PFOS are also very persistent in the human body, and exposures to these chemicals are known to have a number of adverse effects in laboratory animals and humans.
Rhode Island Department of Health	As a result, as people get exposed to PFAS from different sources over time, the level of PFAS in their bodies may lead to adverse health effects. The likelihood of adverse health effects depends on several factors such as the amount and concentration of PFAS ingested as well as the time span of exposure.
Tennessee Department of Environment and Conservation	There is evidence that exposure to PFAS can lead to adverse health outcomes in humans … However, a review of potential human health effects due to PFAS exposure by the Australian Government Department of Health’s Expert Health Panel concluded “there was insufficient evidence of causation between PFAS exposure and any adverse health outcomes.”
Texas Department of State Health Services	PFAS exposure may be associated with increased risk of some adverse effects on human health and may include [list of health effects] … However, these types of health problems can be caused by many different factors including lifestyle, environmental, social, and genetic, and it is difficult to know if PFAS exposure has caused health problems or made them worse.
Utah Department of Environmental Quality	Some, but not all, studies in humans with exposure to PFAS have shown that certain PFAS may [list of health effects].
Vermont Department of Health	Some scientific studies suggest that certain PFAS may affect different systems in the body. Although more research is needed, some studies in people have shown that certain PFAS may [list of health effects].
Virginia Department of Health	Studies in humans and animals show that there may be negative health effects from exposure to certain PFAS. Completely stopping exposure to PFAS is not practical, because they are so common and present throughout the world.
Washington State Department of Health	Current public health recommendations to limit PFAS exposure are typically based on health effects in laboratory animals and findings from observational studies in humans that have been exposed to PFAS. Such studies suggest that higher exposure to certain PFAS may lead to [list of health effects].
Washington State Department of Health – Resources for Health Care Providers	Laboratories capable of processing individual clinical serum samples collected by health care providers are listed below.
Wisconsin Department of Health Services	This research suggests that high levels of certain PFAS may [list of health effects].
**Local Governments / Associations**	
Mariana Islands Water Operator Association	Studies indicate that exposure to PFOA and PFOS over certain levels may result in adverse health effects …
Spokane Regional Health District	Some studies on people exposed to PFOA and PFOS over a long period of time indicate that exposure may [list of health effects].
Town of Natick, MA: Department of Public Works Water/Sewer Division	Some people who drink water containing PFAS6 in excess of the MCL may experience certain adverse effects.

All agency/organization website links and quotations were tested and cross checked on March 14, 2022

Community leaders and scientists have noted that official PFAS health messages to the public and to clinicians are misaligned with the scientific evidence, insufficiently helpful, or even unhelpful [22]. Our review finds that common problems are: 1) failure to distinguish the significance of severely contaminated communities; 2) failure to distinguish levels of evidence for outcomes, with frequent emphasis on doubt that appears to inappropriately encompass all outcomes; 3) weak clinical messaging, including a misdirected focus on the complex legal question of post hoc assessment of causation at the individual level; and 4) no parallel efforts to address the actionable risk reduction/preventive care needs and concerns of those who live in heavily PFAS contaminated communities. Too many documents have odd wording that is either factually indefensible or a diversion from the needs of PFAS contaminated communities.

The US EPA is directly concerned with water contamination. Its site advises consumers about the significance of minimizing the ingested dose including from contaminated water as well as from food. It includes a statement of risk that indicates the health reasons for decreasing exposure.

*“Because certain PFAS are known to cause risks to human health, the most important steps you and your family can take to protect your health is to understand how to limit your exposure to PFAS”* [87].

The site also contains advice about how to reduce doses from food and water, and steps that can be taken to determine if water is contaminated. Unfortunately, it contains limited advice about obtaining the resources to test water for PFAS where water information is not yet available.

The EPA site indicates that the reason to reduce exposure is to reduce risk. However, reasons to avoid exposure or to take other public health action are frequently presented in equivocal or even misleading ways in official documents. Unqualified “may harm,” “may affect,” “possible adverse,” or “could” statements do not provide much information about the reason to take preventive measures, nor encouragement to do so. The Delaware site provides an example, noting that experimental outcomes in some studies can be serious but failing to provide actionable information.

*“Some toxicological studies have found that exposure to these substances can cause serious health effects”* [88].

Sites providing unqualified “may,” “could,” or “some studies” statements give the official entity the comfortable advantage of being correct in all circumstances and the reader the disadvantage of receiving minimal information. Community leaders, policymakers, clinicians, and individuals seeking guidance from official statements can read the language to inappropriately imply across-the-board doubt of consistency or importance for all outcomes, while substantial human population and experimental data consistency exist for a growing list of health outcomes.

A longer list of sites including agencies from New Hampshire, Ohio, and Vermont, provide “may” or “could” statements with a helpful list of relevant outcomes, yet they use language that emphasizes uncertainty for all outcomes. For example, the New Hampshire Department of Health and Human Services (DHHS) communication has this statement:

*“Because of inconsistent and contradictory findings of the effects of PFAS in people, more scientific studies are needed to be sure which health effects, if any, are caused by exposure to PFAS”* [89].

A helpful list of the relevant population outcomes on the New Hampshire site includes kidney and testicular cancer, liver enzymes, cholesterol levels, uric acid increases, and lower immune function, as well as some other outcomes with evidence that may be less consistent. While the New Hampshire document reasonably calls for more research, it unreasonably leaves inconsistency of outcomes as its major message, a conclusion that is not defensible across-the-board. The need for ongoing PFAS research is recognized from past leadership efforts [90] and explicitly endorsed. However, the acknowledged need for more research about all outcomes and the desirability of rigorous examination in no way contradicts the need for health communications to objectively acknowledge and reasonably convey the current scientific evidence. It is not clear why health some official health communications have emphasized doubt for all health outcomes associated with PFAS exposure or what goal that is intended to achieve.

The North Carolina Department of Human Health and Human Services (DHHS) has a “may” statement and a useful list of outcomes, and a generic yet helpful clarification that PFAS risks do not exist alone.

*“Whether or not you develop health problems after being exposed to PFAS depends on how much, how often, and for how long you are exposed, as well as which PFAS you are exposed to. Personal factors including age, lifestyle, and overall health can impact your body's ability to respond to chemical exposures”* [91].

This useful language suggests the presence of susceptible populations and could be tied to affirmative calls for action that invoke patient-clinician partnerships for improved health.

The US National Institute of Environmental Health Sciences (NIEHS) also lists specific outcomes as “possible links,” and emphasizes its research mission as follows:

*“While knowledge about the potential health effects of PFAS has grown, many questions remain unanswered”* [92].

The state of Washington Department of Health (DOH) acknowledges the existence of ongoing research and provides the fundamentally important concept that PFAS health outcomes have parallel data in experimental studies, although it does not emphasize that they show very similar and potentially explanatory findings for some of the experimental outcomes. The language reads as follows.

*“Scientists are still studying how PFAS affect people’s health. Current public health recommendations to limit PFAS exposure are typically based on health effects in laboratory animals and findings from observational studies in humans that have been exposed to PFAS”* [93].

In addition, the Washington DOH health communication provides “may lead to” language for health outcomes that include cholesterol, immune response to vaccines, changes in liver enzymes that indicate liver damage, risk of testicular and thyroid cancer, as well as decreased birth weights and increased risk of thyroid disease [93].

The CDC-funded Pediatric Environmental Health Specialty Units are at academic centers. The unit from US EPA Region 3 helpfully discusses levels of evidence, focusing on “definitive evidence” vs. “compelling evidence” or “strong evidence” as standards of causation, providing an example of strong evidence:

*“The most consistent findings from epidemiology studies are elevated blood serum cholesterol among exposed populations, with strong evidence for a causal relationship between PFOA exposure and elevations in serum lipids”* [94].

This characterization of weight-of-evidence is useful for communicating to patients and clinicians, especially compared to the “sufficient evidence” and “limited evidence” language in scientific documents from which health communications are often derived.

In contrast, The National Health and Medical Research Council of Australia (NHMRC) acknowledges specific cholesterol, renal, and endocrine findings, yet provides a scientifically puzzling blanket assessment concerning the level of evidence for health impact:

*“PFAS has not been shown to cause disease in humans”* [95].

It is at least a frank characterization, however, it is a strange statement and not aligned with modern reviews. Possibly, this statement is meant to be taken in the limited context of the guidance subject only, which is recreational water exposure. However, the Australian Government Department of Health has communicated a similarly puzzling message about all exposure pathways.

*“PFAS has not been proven to cause any specific illnesses in humans”* [96].

Australia’s official health communication further indicated that the associations to lipid abnormalities, which are diagnosable health outcomes, may be due to confounding by diet. Of course, these associations are present across international diets and are clearly seen in multiple large populations afflicted with PFAS water contamination, with a replicable dose response [47, 48]. It is hard to understand why a national department of health would invoke confounding by diet in the face of these data, and difficult for the reader to be clear what is being conveyed about evidence by the choice of the word “proven.” At best, the communication is misleading about the current weight-of-evidence for some outcomes such as abnormal lipid profiles and kidney cancer, and the reader is left uncertain what level and evidence for human and experimental studies is indicated by “proven.”

We also reviewed a shorter but important group of public agency communications intended to assist in discussions between exposed patients and their healthcare providers (Table S1). Clinicians who have a broad scope of duties and substantial time pressure are likely to seek guidance from these sources.

In 2018, North Carolina created a clinician letter to accompany the public communications mentioned above. It prominently discouraged PFAS blood concentration testing in patients even though North Carolina has a wide region of PFAS potable water contamination. Its contents included the following:

*“It remains unclear if these tests would be clinically useful, and it is not possible to connect PFAS test results with clinical outcomes.*

*“It is important to communicate that these tests cannot:*

- *tell them where or how they were exposed to PFAS;*
- *tell them what, if any, health problems might occur, or have occurred, because of PFAS exposure; or, used to guide treatment decisions”* [97].

The 2018 North Carolina guidance was taken off the web and followed by a 2020 letter to clinicians that provided a “some studies” and “researchers working” update concerning outcomes. This update has at least omitted the problematic advice concerning testing [97]. The initial advice to clinicians stated that testing for PFAS exposure does not determine the source. This advice was already known to be misleading where dominant water sources of contamination been identified [98], the pertinent situation in North Carolina and other states where the demand for such testing exists [98]. Individuals who have had their lives disrupted on many levels by PFAS water contamination reasonably want to know about their internal contamination, and, over time, if their personal level of contamination is decreasing.

The state of Washington Department of Health (DOH) and the Association of Public Health Laboratories (APHL) provide helpful, nonjudgmental information concerning the PFAS blood testing, including the difficulty faced by individuals who seek to obtain PFAS testing. The APHL site helpfully contrasts the body burdens that are present in virtually “everyone” with higher body burdens that characterize certain work groups such as firefighters and residents of heavily exposed communities. APHL also specifically mentions cost. The cost estimate for PFAS testing on the APHL site is more pertinent to per capita costs of mass testing of entire communities, underestimating the fiscal barriers to individuals who make seek testing on their own, in the absence of a coordinated community effort.

The most prominent patient-clinician communication documents are from the Agency for Toxic Substances and Disease Registry (ATSDR, an agency of the US Department of Health and Human Services). Multiple state agencies refer interested readers including clinicians to the ATSDR site. In its communication to patients entitled “Talking to Your Doctor about Exposure to PFAS,” ATSDR uses the ambiguous “*some studies*” and “*may affect*” language concerning strength of evidence, leading to advice that patients can use to talk with their doctor(s), and listing some conditions of interest:

“…*some studies in people have shown that certain PFAS may affect growth, learning, and behavior of infants and older children, lower a woman’s chance of getting pregnant, interfere with the body’s natural hormones, increase cholesterol levels, affect the immune system, increase the risk of cancer. If you have any of these conditions and have been exposed to PFAS, you can tell your doctor*” [99].

In its parallel message to clinicians, ATSDR provides two specific suggestions for how clinicians can advise patients about PFAS health outcomes [100]:

A. *“The types of health problems that may be associated with PFAS are also caused by a variety of factors (lifestyle, environmental, social, genetic). It is possible that PFAS contributed to your health problems but there is no way to know if PFAS exposure has caused your illness or made it worse.*

*or*

B. *Based on what we know at this time, there is no reason to think your health problem is associated with exposure to PFAS. Researchers continue to evaluate the potential health risks from PFAS so more may be known in the future”* [100]*.*

Both messages are about post hoc causation. One is indeterminate and the other negative. Neither provides a proactive path. Post hoc causation is a weight-of-evidence topic, but it is hard to understand why ATSDR considers the office visit an appropriate time and place for this complex topic.

Post hoc assignment of causation following toxicant exposure is a difficult problem in a clinical office visit. It is outside the interest and experience of many clinicians, and commonly referred to specialists at tertiary care settings. At best, the clinician advice from ATSDR, if followed, diverts attention from the patient concern about clinical roles in disease prevention and mitigation, and represents a lost opportunity for a public health agency to communicate with clinicians. It is also surprising that ATSDR has one message for all purposes and fails to recognize these discussions will usually occur in areas where there have been high levels of PFAS contamination.

Embedded in the clinician advice is the message “*also caused by a variety of factors (lifestyle, environmental, social, genetic).”* Note that this message from the ATSDR is not framed in terms of susceptibility and risk. Instead, it is a prelude to a discussion of post hoc causation that then dismisses the topic of increased risk – “*but there is no way to know if PFAS exposure has caused your illness or made it worse”* [100].

This too is an unusual focus for a public health agency. Health outcomes attributed to a single environmental risk and that are not complicated by lifestyle, social, or other known risk is a short list, possibly limited to asbestos and malignant mesothelioma, and possibly not even that. Insistence on such outcomes could equally rule out the importance of common toxicant-associated outcomes such as asbestos exposure and lung cancer, which is more common but less specific than asbestos and mesothelioma, or lead exposure and childhood neurobehavioral deficits. Clinicians seeking actionable preventive health care advice can interpret the dismissive tone of the two messages together to imply that there are no health outcomes with sufficient data to justify preventive health concerns about PFAS exposure. In the “Supporting Facts” section of a longer version of its clinician guidance (not designed as a fact sheet), ATSDR doubles down with axiomatic statements that are generally true for chronically persistent environmental toxicants, and mostly unrelated to the reality of environmental epidemiology, calling for a dose with an “expected” outcome rather than discussing the more pertinent topic of increased risk.

*“There is no established PFAS blood level at which a health risk is expected, nor is there a level that is clearly associated with past, current, or future health problems.*

*“Health risks associated with PFAS are not specific to PFAS exposures. These health risks are also influenced by many other environmental, social, or genetic factors”* [100]*.*

The language is again dismissive, but the meaning is either misleading or unclear. The first statement becomes incorrect if the topic is increased risk and overlooks that replicated dose responses are available in the literature for lipid outcomes, and good studies describe dose-response for other outcomes of concern such as kidney cancer [16, 47, 48, 79, 101]. When the tone is generally dismissive, it also is challenging for patients and clinicians to understand what is meant by the broad statement that “these health risks are also influenced by many other environmental, social, or genetic factors” [100]. The statement targets the inevitable contributing role of other risks we share as humans. It is misaligned with the usual patient-clinician concern about personal (or community-level) approaches to risk reduction, and it is unhelpful in PFAS-contaminated communities. In normal public health communications, groups with increased risks would be designated as “susceptible” and their increased needs might be emphasized. Language that dismisses increased risks that have compelling or substantial population and experimental data because there are also comorbid risk factors for similar outcomes, including risks that make the population more susceptible to the specific exposure has happened before. The lead industry historically adapted this approach as a tactic. ATSDR finishes this longer version of its clinician guidance with advice that clinicians should assess risk based on their judgment, including the risks of PFAS, but has nowhere provided clarity about the evidence for health outcomes or approaches to risk reduction:

*“Care of a patient exposed to PFAS may be determined based on the patient’s overall risk factors, family health and environmental exposure histories, patient signs and symptoms, and physical examination”* [100]*.*

A clinician would need to go beyond currently available ATSDR guidance to understand the strength, consistency, and clinical importance of the disease outcome evidence for PFAS, or useful and reasonable steps that can have favorable benefit profiles for early detection and subsequent management of PFAS health outcomes.

In recent open meetings sponsored by the National Academy of Sciences, Engineering, and Medicine (NASEM), residents of affected communities described their own experiences, and in some cases their gratitude when their doctor took the time to look at more than ATSDR guidance. It is expected that at some time in future, NASEM will produce a review of current guidance and provide pathways forward. However, when that occurs, and based on their task, NASEM is more likely to provide analysis that can be interpreted by ATSDR and other cognizant agencies, but without a specific template for succinct guidance. It may be some time before that NASEM deliberations lead to revisions of currently available documents.

Fortunately, there are templates for patient-clinician improvement. Concerning risk, the state of Connecticut Department of Public Health provides a brief discussion of the risks and consistency of findings, adding the helpful concept that dose is important and that experimental evidence supports the population data [102]:

*“The main health concerns from ingestion of PFOS, PFOA, PFNA, PFHxS and related PFAS come from studies in laboratory animals which consistently show effects on the liver and immune system, and on growth, reproduction and fetal development. PFAS can also affect the endocrine and hormonal systems and can disturb blood lipids. Studies of human populations exposed to elevated levels of PFOS, PFOA, PFNA, and PFHxS generally support the effects seen in animals. Some studies have also shown an increased risk for kidney cancer, and at very high exposure levels, for testicular cancer”* [102]*.*

This is one of several agency documents that provide helpful contrast to the documents that treat all outcomes and all exposure concentrations as having equal support or doubt.

The Association of State and Territorial Health Officials (ASTHO), a nongovernment professional organization of government health personnel, provides a clinician message about screening for specific PFAS health outcomes based on evidence:

“*What signs and symptoms should I look for? “There is evidence linking certain PFAS (e.g., PFOA and PFOS) to adverse health effects in humans, with more evidence for some effects than for others. Potential health effects include certain types of cancers, high cholesterol, altered kidney function, impacts on pregnancy and the development of infants and fetuses including preeclampsia, low birth weight, and preterm birth”* [103].

The ASTHO document emphasizes clinician agency and is enabling. It also considers public health approaches that are pertinent to clinicians. It goes on to address the potential for community-level approaches such as biomonitoring, and beneficial primary and secondary prevention approaches such as the importance of addressing comorbid risks in the context of PFAS outcomes and a priori susceptibility. This “teachable moment” and patient-clinician agency approach contrasts with ATSDR’s emphasis on comorbid risks as alternative explanations for post hoc causation.

The Silent Spring Institute, a nonprofit agency dedicated to a safer chemical environment, collaborated with other nonprofit agencies and clinicians and scientists at several universities to provide fact sheets for patients and clinicians. These respond to most commonly posed questions by patients and they have a list of counseling topics for clinicians, both are available on the PFAS-REACH exchange website [104]. These address human and experimental evidence, personal and contaminated community level approaches to reducing exposure, and shared patient-clinician decisions. Uniquely, the site lists commonly used clinically familiar laboratory tests pertinent to PFAS health risks. The goal is to address health outcomes of PFAS exposure and approaches to patient-clinician discussion about risk reduction, cognizant of and respecting the limited time available to busy clinicians, while providing approaches with low additional risk because they emphasize familiar approaches. The PFAS REACH site clarifies that the advice and list of laboratory tests is for patients from heavily impacted communities including residents and workers with high PFAS body burdens.

### Improving official health communications on PFAS

Health communications are products of multiple difficult judgments. In its Technical Guidance for PFAS, the Interstate Technology and Regulatory Council (ITRC) lists for public health professionals both experimental findings and parallel possible human disease links from PFAS exposure. The ITRC clarifies that the list is not exhaustive, and describes the health communication challenge:

*“Due to the evolving science of PFAS, project managers, risk assessors, and risk communicators can also become caught in between those who amplify risk and those who deny risk”* [105]*.*

Only some of the extant PFAS health communications parse that tension between risk amplification and risk denial in a reasonable way. Concerning target audiences, the infrequent choice to specifically characterize the needs and community-wide approaches pertinent to high exposure communities leaves a significant gap that ignores the communities with the most demonstrable needs.

Concerning outcomes, unclarified “may cause” statements likely evolve from the most defensible approach to the inevitable amplification/denial tension in health communications noted by the ITRC. “May cause” and the potentially more misleading “some studies” statements have unfortunately obscured and mischaracterized the very things that patients from heavily contaminated communities and their clinicians want to know. The bland statements do not address the existence of a high or low degree of evidence for increased risk of an outcome and the weight of such evidence across experimental and population studies. Based on current guidance from trusted agencies, how will busy clinicians come to know that for PFAS, the “some studies” statement could be “most” or “nearly all” studies for human outcomes, involving multiple populations around the world, notably those involving adult liver and lipid outcomes, and childhood vaccine responses, or that there is also corroborative experimental evidence for the same outcomes?

Further, there is no reason that modern health communications should censor the growing body of PFAS evidence that has accumulated since the C8 Science Panel made its initial epidemiology appraisal of “probable links” from 2012 to 2014 [106]. These ground-breaking deliberations were and are extremely important, as is the subsequently published peer literature which must also be considered. Too many of the lists of outcomes stop with this list, and ignore a growing body of literature.

Concerning agency roles and regrettable wordsmithing, ATSDR’s emphasis on the difficulties of post hoc causation assignments appears to be a contributing reason that patients in PFAS-contaminated communities believe that their science-based concerns are not being addressed. Such messages may have been intended to be reassuring. Patient and clinician presenters in a recent series of Town Hall meetings hosted by the NASEM PFAS Clinical Guidance Committee [22] identified the approach as the opposite of reassuring to patients and not helpful to clinicians, particularly the language to dismiss patients without providing actionable discussion about clinical prevention of increased risk.

Environmental epidemiology is about evidence. Absence of certainty in the face of different degrees of data supporting causation is not reasonably addressed by pretending that substantial doubt pertains to all health outcomes. Ignoring the corroborating experimental data compounds the problem. Fortunately, there are useful leadership examples including communications from state agencies (such as Connecticut) concerning the understanding of risk, from a professional group (ASTHO) concerning risk and screening, and from nonprofit agencies (ITRC, PFAS REACH in cooperation with Silent Spring Institute) also concerning screening. The latter provides specific examples of laboratory testing for health outcomes and approaches to addressing risks that are familiar to treating clinicians. As agencies seek to improve messages specifically intended for patients and clinicians in PFAS-affected communities, these provide guidance for clinicians and patients and templates for improvement by other trusted organizations.

## Conclusions

Immediate action should be undertaken to review and improve official health communications intended to inform about the risks of PFAS exposure and guide medical decisions. NASEM deliberations are likely to point to needs but may not provide a template. We have therefore shone a spotlight on useful documents whose advantages can be emulated. Goals of improved communications should be to consider the needs of communities with high exposures to PFAS. There is a parallel need for health communications for the much larger group of people with some PFAS exposure, which is almost all of us. These should be distinct from communications to heavily impacted communities.

There is also a compelling need to accurately recognize health outcome evidence, to avoid statements that implausibly treat all outcomes as equally likely or unlikely, or to insist on implausible levels of evidence when compelling or substantial and advancing evidence indicates probable existence of health outcomes. We call on trusted agencies, entities, and organizations to recognize that artificially minimizing and dismissive statements have unintended yet inevitable, problematic uses in multiple domains and directly harm affected communities. Official health communications should also encourage rather than discourage teachable moments when PFAS may interact with comorbid risk factors. Official agencies should recognize rather than discourage patient agency and shared decision-making in patient-clinician interactions. Official agencies should encourage rather than ignore or discourage participation in community-level actions in contaminated areas. Accurate and useful health communications will be a major and important step towards enhancing the role of trusted agencies and promoting healing in PFAS-affected communities.

## Supplementary Information

**Additional file 1.**

## Data Availability

All data generated or analyzed during this study are included in this published article [and its supplementary information files].

## Abbreviations

ASTHO: Association of State and Territorial Health Officials
ATSDR: Agency for Toxics Substances and Disease Registry
CDC: US Centers for Disease Control and Prevention
EPA: US Environmental Protection Agency
ng/mL: Nanograms/mL
PFAS: Per- and polyfluoroalkyl substances
PFHxS: Perfluorohexane sulfonic acid
PFNA: Perfluorononanoic acid
PFOA: Perfluorooctanoic acid
PFOS: Perfluorooctane sulfonic acid
PPAR: Peroxisome proliferator-activated receptors
